# Utilizing Publicly Available Cancer Clinicogenomic Data on CBioPortal to Compare Epidermal Growth Factor Receptor Mutant and Wildtype Non-Small Cell Lung Cancer

**DOI:** 10.7759/cureus.14683

**Published:** 2021-04-25

**Authors:** Chirag Dhar

**Affiliations:** 1 Medicine and Cellular and Molecular Medicine, University of California San Diego, La Jolla, USA

**Keywords:** anti-egfr, cbioportal, lung cancer, cancer genomics

## Abstract

Publicly available clinicogenomic data on platforms such as the cancer BioPortal (cBioPortal.org) allow for efficient analyses by researchers with little or no experience working with Big Data. cBioPortal.org also allows for appropriate statistical testing and downloadable images for easy dissemination of findings. In this study, the cBioPortal.org platform was tested and its utility demonstrated by comparing cases of non-small cell lung cancer (NSCLC) with and without epidermal growth factor receptor gene (*EGFR*) mutations. Patients with *EGFR *mutations were more likely to be female, of Asian ethnicity, never-smokers, and be diagnosed with lung adenocarcinoma. Metastasis to the pleura, pleural fluid, and liver was common in patients with *EGFR *mutant NSCLC. On the other hand, lymph node, brain, and adrenal gland metastases were more common in patients with other mutations. While the median overall survival was about the same in the two groups, progression-free survival was significantly shorter in the *EGFR *mutant group. The mutational landscape was significantly different in the two groups with *EGFR *mutant NSCLCs having a lower mutational burden. Differences in copy number alterations between the two groups were also noted. The descriptive data generated from this study such as age, gender, smoking history, and histological subtype recapitulate findings of other studies on *EGFR *mutant NSCLCs. Further prospective and/or preclinical studies are needed to confirm differences noted in this study. cBioPortal.com queries may be used to supplement clinical/pre-clinical studies or to generate novel hypotheses.

## Introduction

The establishment of cBioPortal.org, a central resource for patient- and sample-level clinicogenomic data in cancer, has allowed for in-depth analyses and comparisons of various cancer subtypes [1,2]. This platform provides fast and efficient analyses and is useful to researchers with little or no experience of working with large datasets. cBioPortal.org also has inbuilt functionality to perform relevant statistical tests and to download “publication quality” images. These outputs may then be used to support small clinical studies, pre-clinical findings, or even to generate novel hypotheses. To test and to demonstrate the utility of this platform, cBioPortal.org was queried for a common lung cancer mutation.

Lung cancers are one of the leading causes of cancer in both men and women and are a major cause of mortality in the American population with an anticipated 135,000 deaths in 2020 [3]. Eighty percent of all lung cancers are further categorized as non-small cell lung cancer (NSCLC) [4]. Major histological subtypes of NSCLC include lung adenocarcinoma, squamous cell carcinoma large cell carcinoma, adenosquamous cell carcinoma, and sarcomatoid carcinoma [5]. Recent advances in the clinicogenomics of lung cancer have uncovered the role of epidermal growth factor receptor (*EGFR*) mutations in a significant proportion of NSCLC patients [6]. *EGFR* is a member of the tyrosine kinase receptor family and plays an important role in cellular growth, proliferation, and signaling. Certain somatic *EGFR* mutations observed in a subset of NSCLC patients cause overamplification leading to constant activation and uncontrolled cell division [5]. Exons 18, 19, 20, and 21 of the *EGFR* gene are often the site of these mutations [7,8]. Some of the commonest *EGFR* mutations include inframe deletions of exon 19 and the exon 21 L858R point mutation [3]. These mutations are present in nearly a third of all lung adenocarcinomas and predict efficacy to *EGFR* tyrosine kinase inhibitors (TKIs) such as gefitinib and erlotinib [9]. Patients receiving TKIs have improved clinical outcomes as compared to those patients that receive conventional chemotherapy [10]. In this retrospective study, the clinicogenomic characteristics of *EGFR* mutant and *EGFR* wildtype NSCLC were compared and contrasted on the cBioPortal platform.

This article was previously posted on a preprint server: https://www.medrxiv.org/content/10.1101/2020.08.07.20170027v3.

## Materials and methods

**Figure 1:**
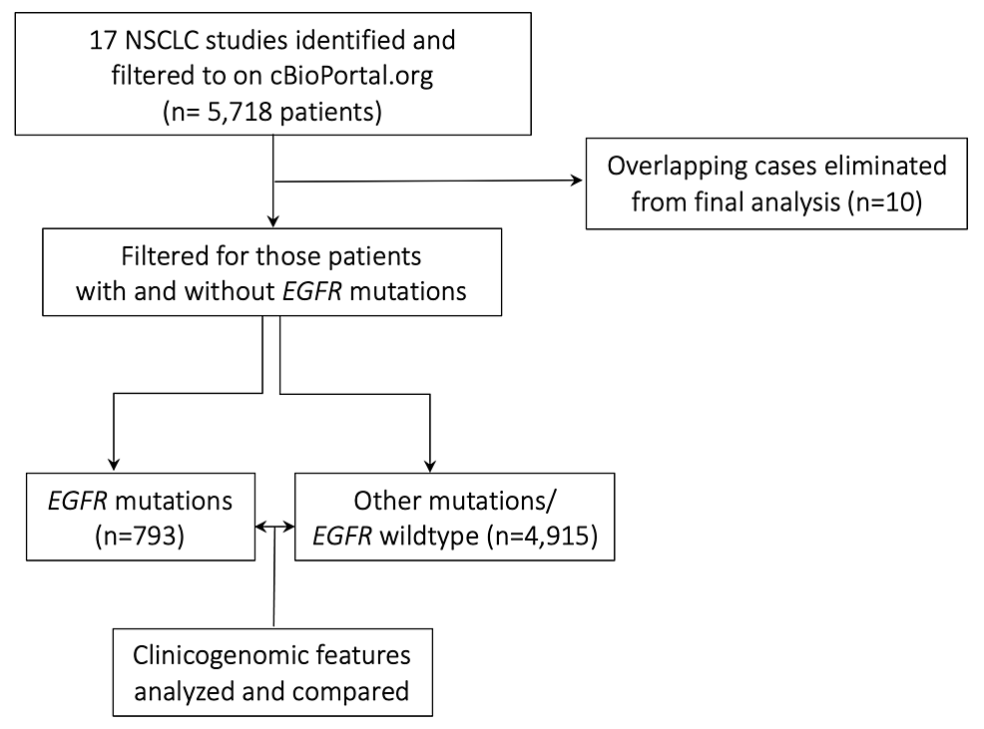
Study schema A number of NSCLC studies (n=17) were identified on cBioPortal.org that included a total of 5,718 patients. These cases were then divided in to those with and without *EGFR* mutations. cBioPortal classified 10 overlapping cases that were present in both groups likely because these patients had contributed multiple samples to these studies. After eliminating these overlapping cases, about 14% (n=793) were found to have *EGFR* mutations and the remaining 86% (n=4,915) were found to have other mutations (*EGFR w*ildtype). These two sub-groups were then compared by utilizing the cBioPortal.org environment and its functions. NSCLC: non-small cell lung cancer; *EGFR*: epidermal growth factor receptor

The following schema (Figure 1) describes the methods of data collection and analysis in this retrospective cohort study. The references to studies utilized are provided here: [11-23].

Briefly, 17 cBioPortal.org NSCLC studies representing 5,718 patients were identified and filtered in September 2020. Of these, 803 were categorized to have *EGFR* mutations while the remaining 4,925 were not. A further analysis on the patient overlap function of cBioPortal.org revealed 10 cases that were present in both groups. These 10 patients possibly represent multiple samples that had different mutational patterns. For the purpose of this study, these overlapping samples were not analyzed. This elimination of overlapping samples was done to prevent the confounding effects of these cases being present in both groups. Also, inclusion of these samples would have biased some results towards the null.

Subsequently, clinicogenomic features of the two subgroups were compared utilizing the various functions available on cBioPortal.org. These analyses included a comparison of survival, staging, mutations, and copy number variations. Appropriate statistical tests/graphical representations as run by cBioPortal.org are included in the results. P- and Q-values where appropriate are mentioned. The phrase “*EGFR* wildtype” and “other mutations” are used interchangeably to represent the sub-group that did not harbor *EGFR* mutations.

## Results

*EGFR* mutations were more common in women, patients of Asian ethnicity, never smokers, and those with lung adenocarcinoma

Clinical characteristics of patients with *EGFR* mutations and those with other mutations (*EGFR* wildtype) were compared and are described in detail in Table 1. The median age of diagnosis was slightly lower in patients with *EGFR* mutations. These patients were more likely to be never-smokers, women, of Asian ethnicity, and diagnosed with lung adenocarcinoma. These findings are in concert with those of other studies [24-28] on *EGFR* mutant NSCLC and suggest this cohort of more than five thousand patients is likely representative of differences seen in individual studies.

**Table 1:** Clinical characteristics of patients in the two subgroups The median age in the *EGFR* group was 64 years (range: 36-92, 25th percentile- 57, 75th percentile- 71) while in the other mutations group it was 67 years (range: 38-93, 25th percentile- 59, 75th percentile- 73) with an absolute p-value by Kruskal Wallis test being 6.25 x 10^-10^ and q-value of 6.07 x 10^-9^. A Chi-squared statistic of 155.708 was obtained for the comparison of sex distribution in the two groups (p-value was <10^-6^). Ethnicity data was available for 280 and 2,047 patients in the two groups with a significantly higher percentage being of Asian/Chinese ethnicity in the *EGFR* group (Chi-squared test statistic was 397.8601, p-value <10^-6^). Smoking histories of 403 and 2,221 patients was available in the two subgroups with never-smokers being significantly higher in the *EGFR* group (Chi-squared test statistic was 296.4889, p-value <10^-6^). 744/805 *EGFR* cases and 3096/4915 other cases were lung adenocarcinoma (Chi-squared test statistic was 2755511, p-value <10^-6^). *all p-values reported to the third decimal place in the table. *EGFR*: epidermal growth factor receptor

Clinical characteristic	* EGFR*	Other mutations	P-value*
Total number of subjects (%)	793 (15.8)	4195 (84.2)	-
Median age at diagnosis in years	64	67	<0.001
Female sex (%)	64.5	41.4	<0.001
Asian/Chinese ethnicity (%)	48.1	7.8	<0.001
Never smokers (%)	38.9	7.9	<0.001
Lung adenocarcinoma (%)	93.8	62.9	<0.001

*EGFR* mutant cancers were more likely to metastasize than cancers with other mutations

**Figure 2:**
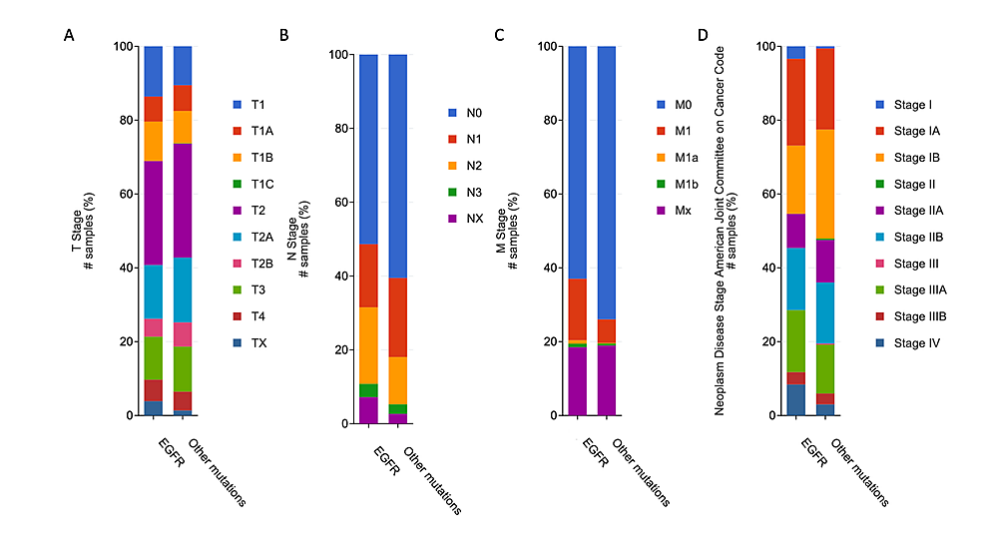
Graphical representation of differences in staging between EGFR mutant NSCLC and tumors with other mutations. A) Differences in T stage: non-significant difference (Chi-squared test p-value 0.393); B) Differences in N stage: non-significant difference (Chi-squared test p-value 0.027); C) Differences in M stage: non-significant difference (Chi-squared test p-value 0.254); D) Differences in American Joint Committee on Cancer Code staging: significant difference with Chi-squared test p-value of 0.00074. *EGFR*: epidermal growth factor receptor; NSCLC: non-small cell lung cancer

Figure 2 shows the differences in TNM and American Joint Committee on Cancer Code staging between *EGFR* mutant NSCLC and tumors with other mutations. *EGFR* mutant cancers were more likely to involve lymph nodes and to metastasize. *EGFR* mutant tumors were about as likely to be diagnosed as stage II, III, and IV as compared to tumors with other mutations.

Metastasis to pleura, pleural fluid, and liver was common in patients with *EGFR* mutant NSCLC, while metastasis to the lymph node, adrenal gland, and brain was more commonly associated with other mutations

**Figure 3:**
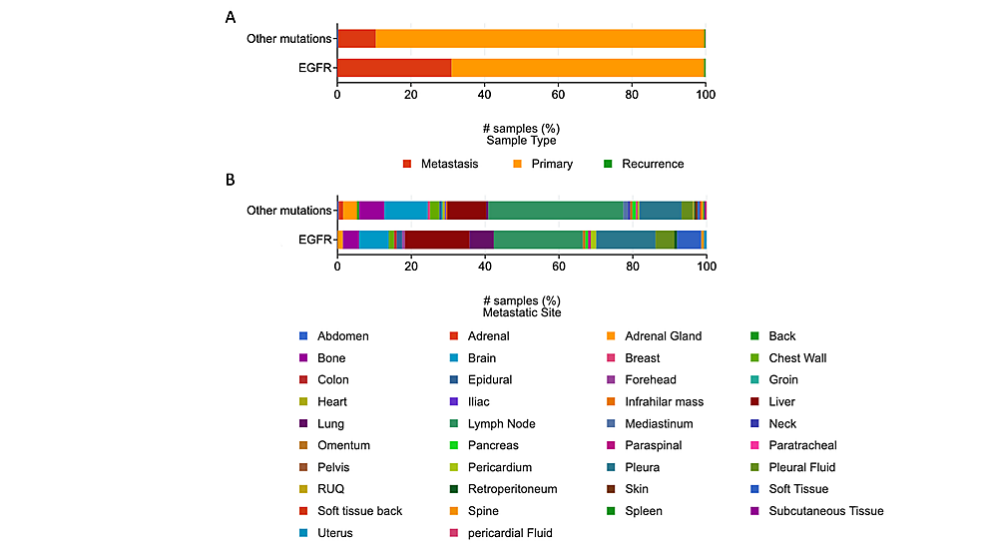
Metastatic samples A) Frequency of metastatic sample collection: metastatic samples were likely to be collected from patients with *EGFR* mutations, though there might be a bias towards obtaining a biopsy in patients with metastatic *EGFR* NSCLC to direct targeted therapy;  B) Frequency of metastatic sample collection by site: there was a significant difference between the two groups (Chi-squared test p-value 0.00641) with EGFR mutant samples more likely obtained from pleura, pleural fluid, and liver while metastatic deposits from the adrenal glands, lymph nodes, and brain were commonly collected from those with other mutations. *EGFR*: epidermal growth factor receptor; NSCLC: non-small cell lunch cancer; RUQ: right upper quadrant

Metastatic samples collected were used as a proxy to indicate frequency and sites of metastases. Metastatic samples were more frequently collected in patients with *EGFR* mutations as indicated by Figure 3A. Collected metastatic samples from pleura, pleural fluid, and liver were common in patients with *EGFR* mutant NSCLC, while metastatic samples from lymph nodes, adrenal gland, and brain were common in patients with other mutations (Figure 3B). These findings suggest a difference where *EGFR* mutant cancers are likely to metastasize.

Overall survival (OS) is comparable in the two groups while progression-free survival (PFS) is shorter in patients with *EGFR* mutations

**Figure 4:**
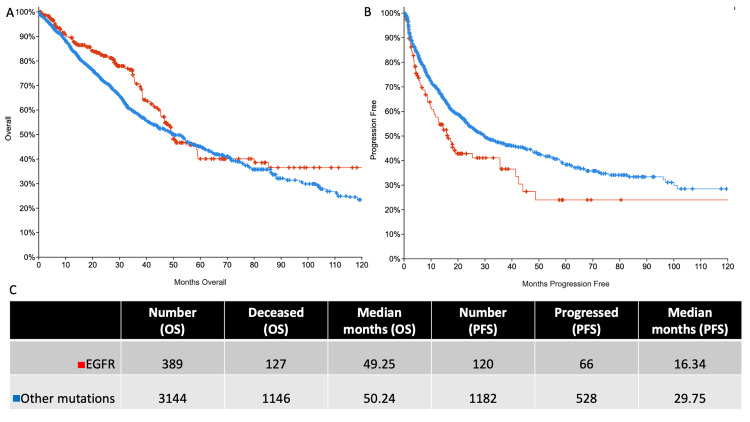
Survival analyses A) Overall survival (OS) Kaplan-Meir curve: logrank test p-value 0.0240; B) Progression-free survival (PFS) Kaplan-Meir curve: logrank test p-value 0.0042; C) Table depicting the number of patients where data was available, number that either died or progressed, and median survival in months. EGFR: epidermal growth factor receptor

Overall survival and PFS were compared in the two groups and Kaplan-Meir curves were generated (Figure 4A, 4B). The OS in both the groups were comparable with *EGFR* mutant cancer patients having a median survival of ~49 months and those with other mutations surviving ~53 months (Figure 4C). The PFS in patients with *EGFR* mutations was significantly shorter than those with other mutations (~16 months versus ~30 months, Figure 4C).

Patients with other mutations have a higher tumor mutation burden and have higher levels of cell-free DNA

**Figure 5:**
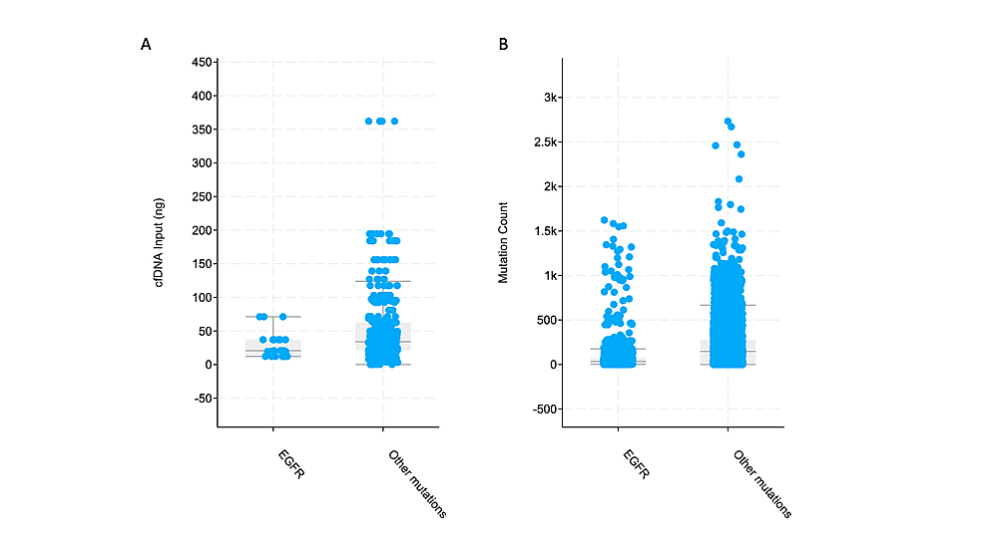
Cell-free DNA and tumor mutation count A) Differences in quantity of cell-free DNA obtained from patients in the two groups: non-significant difference with median quantities being 20.7 ng and 34 ng in *EGFR* and other mutations group respectively (Chi-squared p-value 0.199); B) Differences in tumor mutation count: median mutation count of 35 in *EGFR *group versus 151 in other mutations group (significant difference with Kruskal Wallis test p-value <10^-10^). DNA: deoxyribonucleic acid; cfDNA: cell-free DNA; *EGFR*: epidermal growth factor receptor

The quantity of cell-free deoxyribonucleic acid (DNA) isolated from patients with mutations other than *EGFR* was higher than those with *EGFR* mutations (Figure 5A). Similarly, the tumor mutation burden was higher in patients with other mutations (Figure 5B).

Co-occurrence of mutations and copy number alterations differ in the two groups

**Figure 6:**
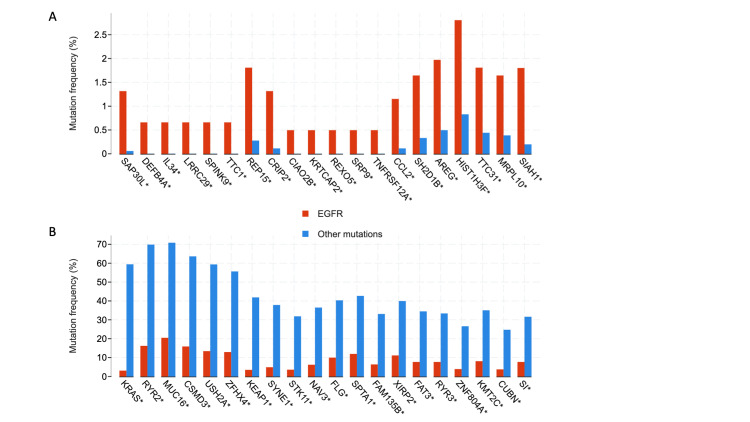
Frequency of commonest co-occurring mutations. *asterisked genes represent significant differences *EGFR*: epidermal growth factor receptor

Commonly co-occurring mutations in the two groups were analyzed. TP53 mutations were amongst the commonest mutations in both groups and were excluded in this analysis. Similarly, as anticipated *EGFR *mutations were most frequent in the *EGFR* mutant group but have been excluded in the analysis. The most frequently co-occurring mutations in the *EGFR* mutant group (with low frequency in the other group) were in *SAP30L, DEFB4A, IL34, LRRC29, SPINK9, TTC1, REP15, CRIP2, CIAO2B, KRTCAP2, REXO5, SRP9, TNFRSF12A, CCL2, SH2D1B, AREG, HIST1H3F, TTC31, MRPL10*, and *SIAH1*. The commonest mutations in patients with mutations other than *EGFR* were *KRAS, RYR2, MUC16, CSMD3, USH2A, ZFHX4, KEAP1, SYNE1, STK11, NAV3, FLG, SPTA1, FAM135B, XIRP2, FAT3, RYR3, ZNF804A, KMT2C, CUBN*, and *SI*. The frequency of occurrence of these mutations is depicted in Figure 6A, 6B.

**Figure 7:**
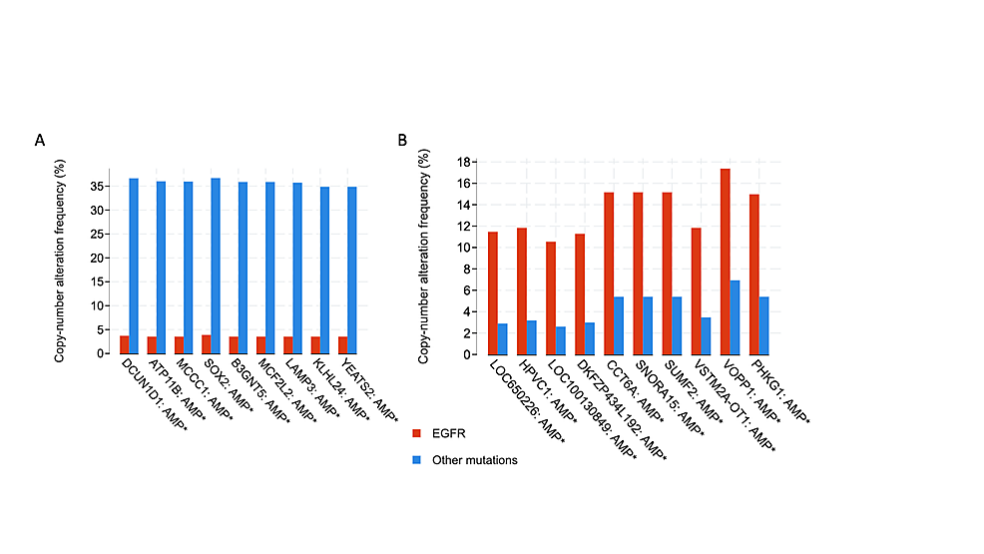
Frequency of various copy number alterations in the two groups *asterisked genes represent significant differences AMP: amplification; EGFR: epidermal growth factor receptor

The commonest copy number alterations were similarly analyzed. As was done with the mutations, *EGFR *amplifications have not been discussed as these are commonly seen in patients with *EGFR* mutations. The commonest amplifications in the *EGFR* group were on *LOC650226, HPVC1, LOC100130849, DKFZP434L 192, CCT6A, SNORA15, SUMF2, VSTM2A-OT1, VOPP1,* and *PHKG1* (Figure 7A). The commonest alterations in the other group were amplifications of *DCUN1D1, ATP11B, MCCC1, SOX2, B3GNT5, MCF2L2, LAMP3, KLHL24,* and *YEATS2* (Figure 7B). 

## Discussion

This retrospective study comparing *EGFR* mutant and *EGFR* wildtype NSCLC confirms the commonest clinical characteristics described in other studies. Patients with *EGFR* mutations were more likely to be female, never-smokers, and of Asian ethnicity. Lung adenocarcinoma was the commonest histological subtype in these patients. These findings being similar to other studies [24-27] pointed to these metadata being representative of differences seen in multiple individual studies. Further, the study showed that there were differences in the staging of cancer between the two groups with *EGFR* mutant cancer being more likely to be diagnosed at a more advanced stage. Metastatic samples collected from the two groups suggest that *EGFR* mutant cancers are more likely to metastasize to the pleura and pleural fluid as well as to the liver. On the other hand, NSCLC tumors harboring other mutations were more likely to metastasize to lymph nodes, the brain, and the adrenal glands. Likely due to these differences in staging and a higher likelihood of metastasis in the *EGFR* mutant group, the progression-free survival was shorter in patients with *EGFR* mutations. Yet, the difference in median overall survival was only 1 month. These findings are striking in light of targeted therapies for *EGFR* mutant cancers with *EGFR* TKIs and are strongly suggestive of rapid development of resistance to these therapies [29]. These can occur in the context of mutations such as T790M on exon [5].

At the genomic level, the tumor mutation burden was higher in *EGFR* wildtype patients. This finding is anticipated as these cancers may lack specific driver mutations and instead rely on multiple mutations for their transformation from normal tissue to cancer. The quantity of cell-free DNA, though not significantly different in the two groups, was lower in patients with *EGFR* mutations. The mutation and copy number alteration landscape were also different in the two groups and the commonest genes that were mutated or amplified are listed in the results section. These unique genomic signatures may be successfully exploited to predict *EGFR* TKI resistance early if confirmed in independent biomarker studies and/or cell-free DNA studies [30]. Prospective studies may show that some of these genomic features are present at the time of primary biopsy itself. Additionally, “bedside to bench” studies can be used to confirm these findings in cell lines and/or animal models which in turn could lead to studies on alternate “druggable” pathways.

Studies conducted on cBioPortal such as this one are limited by their retrospective design and are therefore likely to have a degree of convenience sampling. The effects of this error were likely minimal as the clinical characteristics of the two groups were similar to that seen in other prospective studies (age, sex, ethnicity, and smoking history were consistent with what has been reported in the literature). Additionally, there were limitations in the quality of data. For example, 6 of 17 included studies did not provide copy-number variation data. It is also possible that endpoints such as progression-free survival were calculated differently in the various studies included in these analyses. Despite filtering to NSCLC studies, a small number of lung cancers other than NSCLC (<0.5%) also crept through the filters. This number was very small and likely did not alter the results significantly. cBioPortal data does not include specific treatment data and it is possible that a bias towards the null may have occurred in the overall survival data. Other studies have shown better outcomes in patients with *EGFR* mutations when treated with *EGFR* TKIs [7,10]. Another limitation that pertains to cBioPortal is the inability to run multivariate analysis to identify confounding factors and to adjust for these. cBioPortal does allow one to download the metadata and perform these analyses using other statistical software such as R or SPSS. For the convenience of this study, NSCLC was broadly divided into two groups: one for patients with *EGFR* mutations and the other for patients with wildtype *EGFR*. The second group is an oversimplification and includes a gamut of different mutations including some driver mutations such as *KRAS*. Finally, despite showing clear differences in the mutation and copy number alteration landscape in the two groups, this study does not clearly define differences in the molecular mechanisms.

## Conclusions

Such studies on the clinicogenomic features of NSCLC and other cancers on cBioPortal are likely to throw light on possible new “druggable” targets. Additionally, hypotheses may be drawn from these studies and taken back to the “bench” to understand specific molecular mechanisms such as resistance to *EGFR* TKIs or the role of cell-free DNA. Future prospective studies and clinical trials are likely to include genomic level and transcriptomic analyses to draw broader clinicogenomic conclusions and lead to significant advances in the management of NSCLC.
